# Computational study of effect of hybrid nanoparticles on hemodynamics and thermal transfer in ruptured arteries with pathological dilation

**DOI:** 10.1038/s41598-024-64356-2

**Published:** 2024-06-11

**Authors:** Azad Hussain, Muhammad Bilal Riaz, Muhammad Naveel Riaz Dar, Rimsha Kanwal, Lubna Sarwar, Adil Jhangeer

**Affiliations:** 1 Department of Mathematics, University of Gujrat, Gujrat, 50700 Pakistan; 2 IT4Innovations, VSB–Technical University of Ostrava, Ostrava, Czech Republic; 3 Department of Computer Science and Mathematics, Lebanese American University, Byblos, Lebanon

**Keywords:** Hybrid nanoparticles (silver and gold), 3-D simulation, BFD, Sidewall ruptured dilatation, Engineering, Mathematics and computing

## Abstract

The intended research aims to explore the convection phenomena of a hybrid nanofluid composed of gold and silver nanoparticles. This research is novel and significant because there is a lack of existing studies on the flow behavior of hybrid nanoparticles with important physical properties of blood base fluids, especially in the case of sidewall ruptured dilated arteries. The implementation of combined nanoparticles rather than unadulterated nanoparticles is one of the most crucial elements in boosting the thermal conduction of fluids. The research methodology encompasses the utilization of advanced bio-fluid dynamics software for simulating the flow of the nanofluid. The physical context elucidates the governing equations of momentum, mass, momentum, and energy in terms of partial differential equations. The results are displayed in both tabular and graphical forms to demonstrate the numerical and graphical solutions. The effect of physical parameters on velocity distribution is illustrated through graphs. Furthermore, the study’s findings are unique and original, and these computational discoveries have not been published by any researcher before. The finding implies that utilizing hybrid nanoparticles as drug carriers holds great promise in mitigating the effects of blood flow, potentially enhancing drug delivery, and minimizing its impact on the body.

## Introduction

The prevalence of cerebral aneurysms a common vascular disease, ranges from 20 to 50 per 1000 people^1,2^. Aneurysm rupture is a catastrophic and potentially fatal event caused by the rupture of a weakened aneurysm wall, leading to an outpouring of blood into the area surrounding the aneurysm. When evaluating cerebral aneurysm rupture risk, the primary criteria are familial and morphological aneurysm features^3^. The morphological parameters, and occasionally a combination of morphological and hemodynamic parameters can be used to predict aneurysm rupture^4^. Many factors can affect the development of an aneurysm, but two major ones are biochemical (the secretion of growth factors as a result of intraluminal thrombosis) and biomechanical (such as wall shear stress and relative residence time)^5^. Although aneurysms larger than 10 mm are thought to be harmful, research has revealed that a significant portion of ruptured aneurysms are, in fact, smaller than 10 mm^6–10^. Carter et al. examined 854 ruptured aneurysms and 819 unruptured aneurysms and discovered that, in decreasing order of average aneurysm size, the posterior communicating artery, basilar bifurcation, middle cerebral artery bifurcation, poster inferior cerebellar artery, and "distal" locations were the sites of ruptured lesions^11^.

Aneurysms are a highly researched topic due to their intricate nature, and investigations have centered on a variety of factors that shape their behavior, chiefly concerning the formation, development, and rupture of aneurysms. This includes clinical studies, experimental fluid dynamics, and computational and numerical studies that provide an understanding of aneurysm behavior utilizing patient-specific models and flow scenarios. Clinical research concentrated on identifying crucial morphological stages of aneurysm formation and rupture. The analyst investigates that, male patients’ aneurysm sizes are 9.2 and 7.4 mm respectively for female patients^12–14^. Age also affects the growth and rupture of aneurysms^15^. Early research on aneurysms conducted by Ferguson indicated that turbulence and intra-aneurysmal pressure may weaken and grow the aneurysm wall, increasing the risk of rupture^16^. Then it was proposed that aneurysms could develop through hemodynamically stressed degenerative lesions^17^ or through high-flow fluctuations that could promote growth or rupture^18^. Researchers have used computational studies to simulate various aneurysm flow scenarios and construct realistic aneurysm geometries using data from MRI scans of individual patients^19–23^.

The advancement of heat transmission has long piqued scientists’ interest. Researchers from all over the world have devoted a lot of time to figuring out how much thermal enhancement nanofluids have. Choi^24^ was the first to create nanofluids, which are artificial colloids composed of a base fluid and nanoparticles. Nanoparticles, which are particles with diameters much smaller than 100 nm, have thermal conductivities that are often orders of magnitude greater than those of the basic fluids. The base fluids’ ability to transport heat is considerably improved by the addition of nanoparticles^25–27^. The basic fluids can be water, oils and lubricants, biofluids, polymeric solutions, and other typical liquids. Organic liquids (such as ethylene, trimethylene-glycols, refrigerants, etc.) are also acceptable. Due to their distinct interaction with matter, nanoparticles (NPs) are synthetic materials with a wide range of uses in biomedicine^28^. In optical, photoacoustic, and MRI imaging, NPs can be used as contrast agents. They can also be used in the drug delivery process as carriers that can improve the therapeutic impact by lengthening circulation instances, protecting carried drugs from degradation, and enhancing tumor uptake. Hybrid nanofluids are a brand-new type of nanofluid that can be created by suspending hybrid (composite) nanoparticles in base fluid together with multiple types of nanoparticles (two or more types). A hybrid substance combines the physical and chemical properties of various materials at the same time and offers these properties in a homogenous state. The properties of these composites have been extensively studied^29–35^, and hybrid materials containing carbon nanotubes (CNTs) have been applied in nanocatalysts, electrochemical sensors, biosensors, and other applications. However, the application of these hybrid nanomaterials in nanofluids has not yet been established. There has been very little research on hybrid nanofluids, but both theoretical and experimental work is still ongoing. In the presence of moderate stenosis lesions, Ahmed and Nadeem^36^ studied the research on many types of nanoparticles, including copper (Cu), titanium (TiO2), and aluminum (Al_2_O_3_).

In this research, we investigate the effects of hybrid nanoparticles on blood flow in impaired arteries with side wall ruptured aneurysms. Previous research does not explore this topic using 3D numerical modeling, which is a novel and advanced method. Therefore, we conduct this research to fill the gap in the literature. We use blood as a base fluid and consider it a Newtonian fluid due to its characteristics in large cavities such as arteries. We analyze the effects of the gold and silver nanoparticles on blood flow by inserting them into a ruptured and dilated area. We use mathematical models to explore how to improve blood flow and prevent further complications.

## Mathematical model and problem description

**Figure 1:**
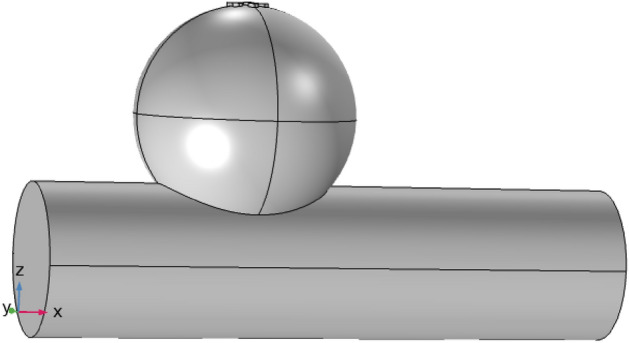
The geometry of the ruptured sidewall pathological dilatation.

In this study, we investigate the flow of a Newtonian, incompressible fluid through an artery with a ruptured bulging on the upper side of the wall, as shown in Fig. 1. A fluid flow path parallel to the z-axis and analogous to the r-axis is generated using a cylindrical flow geometry with a constant radius. This enabled the investigation of the effects of axial and radial variations in the fluid characteristics and dynamics. The present study investigates the causes and consequences of flow instability. The results of this study are then applied to investigate the effects of the geometrical structure of the ruptured aneurysm on the flow of blood. The three-dimensional artery has a width and height of 0.09 and 0.7 m, respectively. The cylinder represents the main artery, and the bulging indicates the location and size of the rupture. The occurrence of a blood vessel wall rupture at the apex of an aneurysm results in the leakage of blood from the artery. If it is not treated right away, this rupture could lead to serious problems like a stroke or even death. Silver and gold particles are added, which strengthened the artery and fixed the burst spot. The cylindrical geometry model is displayed below.

The set of basic equations for blood flow through a dilated artery can be described mathematically as follows:

1 $$\rho \frac{{\partial {\mathbf{v}}}}{\partial t} + \rho \left( {{\mathbf{v}}.\nabla } \right){\mathbf{v}} = \nabla .\left[ { - p{\mathbf{I}}\left] { + \nabla } \right[{\varvec{K}}} \right] + {\varvec{F}}.\user2{ }$$

where $${\mathbf{K}} = {\upmu }(\nabla {\mathbf{v}} + \left( {\nabla \left( {\mathbf{v}} \right)} \right)^{{\text{T}}} )$$.

2 $$\rho \nabla \cdot \left( {\mathbf{v}} \right) = 0,\;\left( {{\text{Incompressible}}\;{\text{flow}}} \right)$$

3 $$\rho C_{p} \frac{{\partial {\text{T}}}}{\partial t} + \rho C_{p} {\mathbf{v}} \cdot \nabla {\text{T}} + \nabla \cdot {\varvec{q}} = Q_{p} + Q_{vd} + Q.$$

The terms below constitute Eq. (3).

$${\varvec{q}} = - k\nabla T,\;Q = 0, Q_{vd} = \tau \cdot \nabla v, Q_{p} = \alpha_{p} T\left( {\frac{\partial p}{{\partial t}} + v\nabla p} \right), \alpha_{p} = - \frac{1}{p}\frac{\partial p}{{\partial t}}.$$

where $$\tau = - pI + \mu A_{1}$$ and $$trace\left( {\tau .\nabla {\text{v}}} \right) = \tau .\nabla {\text{v}}$$.

The geometry boundary condition is as follows:

*Lower and upper walls*: The no-slip condition is considered in this study. This presumption is represented mathematically as:

4 $$u_{1} = 0,{ }u_{3} = 0.{ }$$

*Geometry Inlet*: The blood flow rate is designed to resemble an artery’s entry. By adjusting the stream’s cross-sectional area and inflow rate, the blood volume can be changed. The state of the inlet boundary is described below:

5 $${\varvec{v}}\left( {{\varvec{r}},{\varvec{z}},{\varvec{t}}} \right) = v_{0} {\varvec{n}}.$$

*Geometry Outlet*: We accurately used pressures from the blood flow model at the outflow in the exit to increase the simulation’s accuracy. This outlet is located across from the inlet where the blood left the artery.

6 $$\left[ { - p{\mathbf{I}} + {\mathbf{K}}} \right]{\mathbf{n}} = - \hat{p}_{0} {\mathbf{n}},$$

$$\hat{p}_{0} < p_{0} .$$

In this instance, backflow suppression or the typical internal artery pressure is assumed to be 13,000 Pa.

*The Thermal Insulation Equation*: The geometric borders are all thermally insulated. Additionally, the following is the thermal insulation equation:

7 $$- {\mathbf{n}}. {\mathbf{q}} = { }0.$$

## Numerical simulation

For a particular velocity field, the governing equations for energy, momentum, and mass are given.

$$V = \left( {u_{1 } \left( {r,\theta ,z,t} \right), u_{2} \left( {r,\theta ,z,t} \right), u_{3} \left( {r,\theta ,z,t} \right)} \right)$$.

### Continuity equation

8 $$\frac{{\partial u_{1 } }}{\partial r} + \frac{1}{r}u_{1 } + \frac{1}{r}\frac{{\partial u_{2 } }}{\partial \theta } + \frac{{\partial u_{3 } }}{\partial z} = 0.$$

### Momentum equations

9 $$\rho_{hnf} \left( { \frac{{\partial u_{1 } }}{\partial t} + u_{1 } \frac{{\partial u_{1 } }}{\partial r} + \frac{{u_{2 } }}{r} \frac{{\partial u_{1 } }}{\partial \theta } - \frac{{u_{2 }^{2} }}{r} + u_{3 } \frac{{\partial u_{1 } }}{\partial z}} \right) = - \frac{\partial p}{{\partial r}} + \frac{1}{r}\frac{{\partial \left( {rS_{rr} } \right)}}{\partial r} + \frac{1}{r}\frac{{\partial \left( {S_{r\theta } } \right)}}{\partial \theta } - \frac{{S_{\theta \theta } }}{r} + \frac{{\partial \left( {S_{rz} } \right)}}{\partial z},$$

10 $$\rho_{hnf} \left( { \frac{{\partial u_{2 } }}{\partial t} + u_{1 } \frac{{\partial u_{2 } }}{\partial r} + \frac{{u_{2 } }}{r} \frac{{\partial u_{2 } }}{\partial \theta } - \frac{{u_{1} u_{2} }}{r} + u_{3 } \frac{{\partial u_{2} }}{\partial z}} \right) = - \frac{1}{r}\frac{\partial p}{{\partial \theta }} + \frac{1}{{r^{2} }}\frac{{\partial \left( {r^{2} S_{\theta r} } \right)}}{\partial r} + \frac{1}{r}\frac{{\partial S_{\theta \theta } }}{\partial \theta } + \frac{{\partial S_{\theta z} }}{\partial z},$$

11 $$\rho_{hnf} \left( { \frac{{\partial u_{3 } }}{\partial t} + u_{1 } \frac{{\partial u_{3 } }}{\partial r} + \frac{{u_{2 } }}{r} \frac{{\partial u_{3 } }}{\partial \theta } + u_{3 } \frac{{\partial u_{2} }}{\partial z}} \right) = - \frac{\partial p}{{\partial z}} + \frac{1}{r}\frac{{\partial \left( {rS_{rz} } \right)}}{\partial r} + \frac{1}{r}\frac{{\partial \left( {S_{\theta z} } \right)}}{\partial \theta } + \frac{{\partial \left( {S_{zz} } \right)}}{\partial z}.$$

where $$S = \left( {grad\left( V \right) + \left( {grad\left( V \right)} \right)^{T} } \right).$$

### Energy equation

12 $$\left( {\rho C_{p} } \right)_{hnf} \left( {\frac{\partial T}{{\partial t}} + u_{1 } \frac{\partial T}{{\partial r}} + \frac{{u_{2 } }}{r}\frac{\partial T}{{\partial \theta }} + u_{3 } \frac{\partial T}{{\partial z}}} \right) = {\text{k}}_{hnf} \left[ {\frac{1}{r}\frac{\partial }{\partial r}\left( {r\frac{\partial T}{{\partial r}}} \right) + \frac{1}{{r^{2} }}\frac{{\partial^{2} T}}{{\partial \theta^{2} }} + \frac{{\partial^{2} T}}{{\partial z^{2} }}} \right] + \mu_{hnf} \varphi .$$

wherever $\varphi = 2\left( { \frac{{\partial u_{r } }}{\partial r}} \right)^{2} + 2\left( {\frac{1}{r} \frac{{\partial u_{\theta } }}{\partial \theta } + \frac{{u_{r } }}{r}} \right)^{2} + 2\left( { \frac{{\partial u_{z} }}{\partial z}} \right)^{2} + \left( { \frac{{\partial u_{\theta } }}{\partial r} - \frac{{u_{\theta } }}{r} + \frac{1}{r} \frac{{\partial u_{r } }}{\partial \theta }} \right)^{2} + \left( {\frac{1}{r} \frac{{\partial u_{z } }}{\partial \theta } + \frac{{\partial u_{\theta } }}{\partial z}} \right)^{2} + \left( { \frac{{\partial u_{r } }}{\partial z} + \frac{{\partial u_{z } }}{\partial r}} \right)^{2} .$ Since we use a velocity field of $v=0$ in these equations and the flow is independent of an angle, the (8)–(12) becomes:

13 $$\frac{{\partial u_{1 } }}{\partial r} + \frac{{u_{1} }}{r} + \frac{{\partial u_{3 } }}{\partial z} = 0,$$

14 $$\left( { \frac{{\partial u_{1 } }}{\partial t} + u_{1 } \frac{{\partial u_{1 } }}{\partial r} + u_{3 } \frac{{\partial u_{1 } }}{\partial z}} \right) = - \frac{1}{{\rho_{hnf} }}\frac{\partial p}{{\partial r}} + \nu_{hnf} \left( \frac{2}{r} \right.{ }\frac{{\partial u_{1 } }}{\partial r} + 2\frac{{\partial^{2} u_{1} }}{{\partial r^{2} }}\left. { - \frac{{2u_{1} }}{{r^{2} }} + \frac{{\partial^{2} u_{3} }}{\partial z\partial r} + \frac{{\partial^{2} u_{1} }}{{\partial z^{2} }}} \right),$$

15 $$\frac{\partial p}{{\partial \theta }} = 0,$$

16 $$\left( { \frac{{\partial u_{3 } }}{\partial t} + u_{1 } \frac{{\partial u_{3 } }}{\partial r} + u_{3 } \frac{{\partial u_{3} }}{\partial z}} \right) = - \frac{1}{{\rho_{hnf} }}\frac{\partial p}{{\partial z}} + \nu_{hnf} \left( {\frac{1}{r}\frac{{\partial u_{3 } }}{\partial r} + \frac{{\partial^{2} u_{3} }}{{\partial r^{2} }} + \frac{1}{r}\frac{{\partial u_{1} }}{\partial z} + \frac{{\partial^{2} u_{1} }}{{\partial z^{2} }} + 2\frac{{\partial^{2} u_{3} }}{{\partial z^{2} }}} \right),$$

17 $$\left( {\rho C_{p} } \right)_{hnf} \left( {\frac{\partial T}{{\partial t}} + u_{1 } \frac{\partial T}{{\partial r}} + u_{3} \frac{\partial T}{{\partial z}}} \right) = {\text{k}}_{hnf} \left( {\frac{1}{r}\frac{\partial T}{{\partial r}} + \frac{{\partial^{2} T}}{{\partial r^{2} }} + \frac{{\partial^{2} T}}{{\partial z^{2} }}} \right).$$

where T stands for the absolute temperature, $\nu_{hnf}$ stands for the kinematic viscosity of the nanofluids, and $\rho_{hnf}$ represents the density. The nanoparticles’ particular heat capacity, as well as thermal conductivity, are $\left( {\rho C_{p} } \right)_{hnf}$ and ${\text{k}}_{hnf}$, respectively.

Thermophysical properties of hybrid nanoparticles include the following^37^.

18 $$\left. {\begin{array}{*{20}l} {\rho_{hnf} = \left( {1 - \phi_{2} } \right)\left( { \left( {1 - \phi_{1} } \right)} \right.\rho_{f} + \phi_{1} \rho_{{s_{1} }} ) + \phi_{2} \rho_{{s_{2} }} ,} \hfill \\ {\left( {\rho C_{p} } \right)_{hnf} = \left( {1 - \phi_{2} } \right)\left( {\left( {1 - \phi_{1} } \right)\rho C_{p} } \right)_{f} + \phi_{1} \left( {\rho C_{p} } \right)_{{s_{1} }} ) + \phi_{2} \left( {\rho C_{p} } \right)_{{s_{2} }} ,} \hfill \\ {\mu_{hnf} = \frac{{\mu_{f} }}{{\left( {1 - \phi_{1} } \right)^{2.5} \left( {1 - \phi_{2} } \right)^{2.5} }}, } \hfill \\ {\frac{{K_{hnf} }}{{K_{f} }} = \left\{ {\frac{{k_{{s_{1} + }} 2k_{f} - 2\phi_{1} \left( {k_{f} - k_{{s_{1} }} } \right)}}{{k_{{s_{1} + }} 2k_{f} + \phi_{1} \left( {k_{f} - k_{{s_{1} }} } \right)}} \times \frac{{k_{{s_{2} + }} 2k_{nf} - 2\phi_{2} \left( {k_{nf} - k_{{s_{2} }} } \right)}}{{k_{{s_{2} + }} 2k_{nf} + \phi_{2} \left( {k_{nf} - k_{{s_{2} }} } \right)}}} \right\}. } \hfill \\ \end{array} } \right\}$$

## Computational mesh

**Figure 2:**
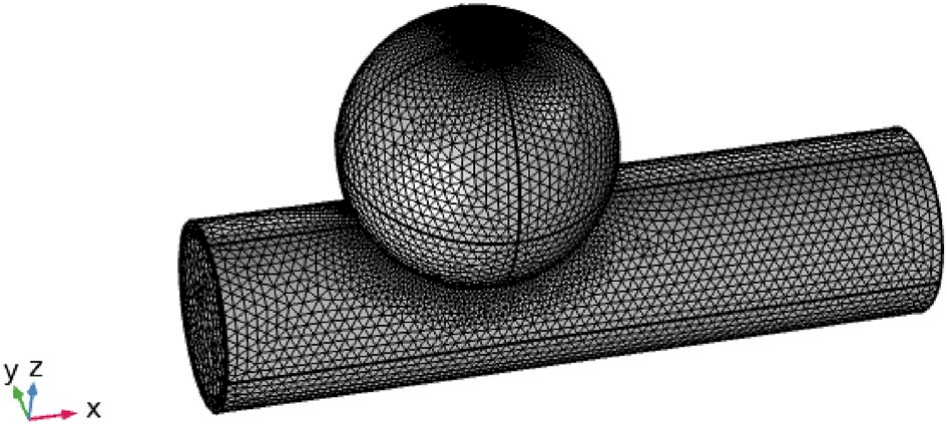
Flow geometry of a ruptured sidewall dilatation using a fine element size mesh.

The computational mesh defines a digital depiction of a physical entity with interconnected nodes and parts. In several technical fields, it is used to express real-world objects. A computational mesh is used in fluid dynamics to examine a fluid’s physical characteristics, including its pressure, velocity, and temperature. The mesh can be used to model how a fluid behaves in various situations, such as when it is flowing through a pipe or around an obstacle. By using a finite component mesh, the accuracy of the solution and the total number of iterations can be used to gauge how accurate and effective the solution is. Additionally, the mesh may be utilized to represent the shape of the object and solution field. We employ a small-element mesh in this shape. Since the elements are significantly smaller and can more accurately represent the specifics of the material’s behavior, it enables a more precise examination of the substance. Furthermore, a finer element mesh can deliver more precise boundary constraints, which can aid in lowering the number of problem-solving iterations. According to Fig. 2, the geometries close to the hole had a smaller mesh size and less rectification than the regions further away from the rupture. Mesh statistics are shown in Table 2, whereas mesh size is shown in Table 3.

Finite Element Method solvers are primarily utilized for structural mechanics and heat conduction problems. They rely on the Galerkin weighted residuals method or Galerkin Finite Element Method. These approaches prove highly effective as they lead to symmetrical stiffness matrices due to the consideration of position and thermal equilibrium situations^39^. This method discretizes the domain into a mesh of finite elements and approximates the solution of the governing equations by a linear or higher-order combination of shape functions defined on each element^40^. The Finite Element Method handles arbitrary geometries, boundary conditions, and nonlinearities with high accuracy and flexibility. The physics at the level of each element is estimated by this approximation. Then, the contributions of all elements are combined in a big sparse matrix equation system. Different solvers, such as MUMPS, PARDISO, are used to solve this system^41^. The advantage of the Finite Element Method lies in its mathematical robustness, making it a powerful approach. Additionally, Finite Element Method can utilize higher-order functions for approximating the solution within each element^42^. A three-dimensional blood flow model of the human heart is developed, and the numerical solution is obtained using the computational fluid dynamics technique^43^. By employing this method in conjunction with computational fluid dynamics, we create 3D numerical models of impaired arteries with side wall ruptured aneurysms and analyze the impact of introducing gold-silver hybrid nanoparticles into the blood flow. This novel approach enables us to explore potential improvements in blood flow dynamics and assess the prevention of further complications. The results of our research hold promise for advancing the understanding and treatment of vascular disorders, particularly in the context of aneurysms.

## Computational results and discussion

In this study, we used 3D numerical modeling to examine the impact of hybrid nanoparticles on blood flow in obscured arteries with a side wall pathological dilatation. The main purpose of this study was to evaluate the simulation’s results regarding velocity, temperature, and pressure in the artery after the insertion of hybrid nanoparticles (silver and gold). The blood’s heat capacity, density, thermal conductivity, and dynamic viscosity were altered by the hybrid nanoparticle infusion, which had an effect on the simulation results. At various points throughout the length of the artery, we made a cross-section in the $XZ$ direction with $y = 0$ and discussed it at different times. Table 1 shows thermos-physical properties of blood base fluid and silver and gold nanoparticles. Tables 2 and 3 depicts the mesh statistics and size respectively. Table 4 describes variation of velocity and pressure in line graph.

**Table 1:** The thermophysical properties of a nanofluid composed of blood as a base fluid and silver and gold nanoparticles^38^.

Property	Heat capacity (J K^−1^ kg^−1^)	Thermal conductivity (Wm^−1^ k^−1^)	Dynamic viscosity (Mm^−2^ s)	Density (kg m^−3^)
Blood	3746	0.52	0.003	1063
Gold (Au)	129	310	0.00464	19,300
Silver (Ag)	235	429	0.005	10,500

**Table 2:** Mesh dimensions are described as.

Characteristics	Values
Number of elements	303,117
overall triangles	17,350
Mesh apexes	64,923
Pyramids	936
Minimum element quality	0.1143
Edge elements	1000
Average element value	0.6792
overall quads	192
Tetrahedrons	272,613
Element volume fraction	4.859E − 5
Mesh capacity	0.02638 m^3^
Prisms	29,568
Vertex elements	72

**Table 3:** The mesh’s size is described as.

Element size parameters	
Maximum size of an element	0.0169 m
Minimum element size	0.0032 m
Resolution of narrow regions	0.8
Curvature factor	0.06
Maximum element growth rate	1.13

**Table 4:** Variation of velocity and pressure in a line graph.

Time (t)	Maximum velocity (ms^−1^)	Minimum velocity (ms^−1^)	Maximum pressure (pa)	Minimum pressure (pa)
0.4 s	0.0820	0.063	13,120	13,000
0.8 s	0.085	0.08	13,075	13,000
1.2 s	0.087	0.083	13,060	12,990
2.4 s	0.094	0.082	13,040	13,000

### The velocity contour profile

**Figure 3:**
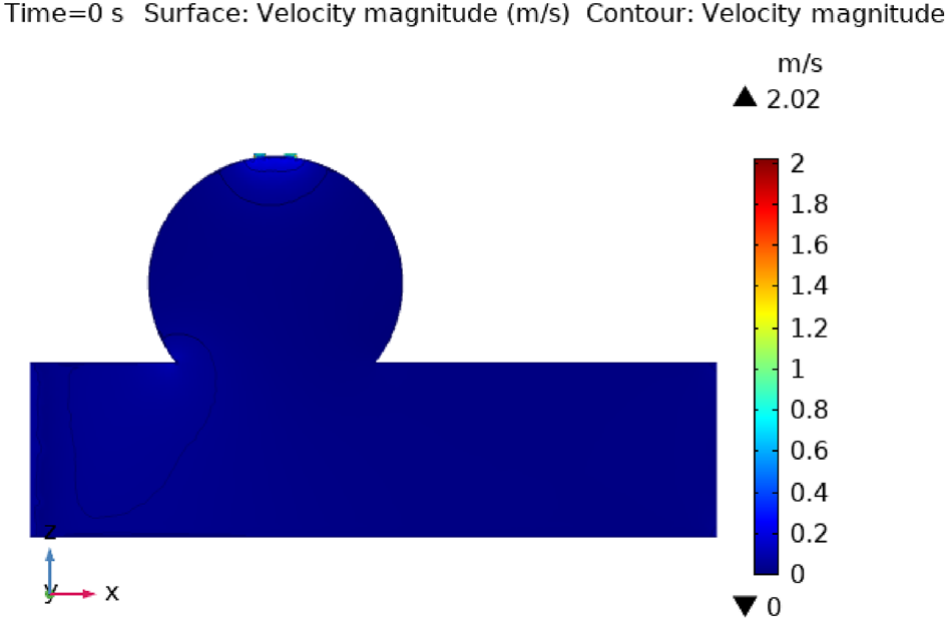
The velocity contour of the blood flow at time = 0 s.

**Figure 4:**
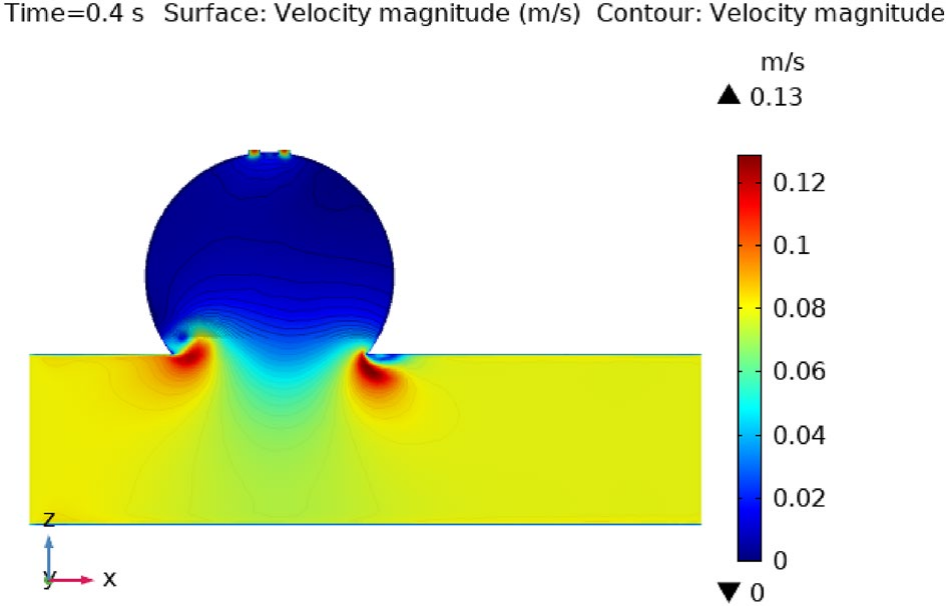
The velocity contour of the blood flow at time = 0.4 s.

**Figure 5:**
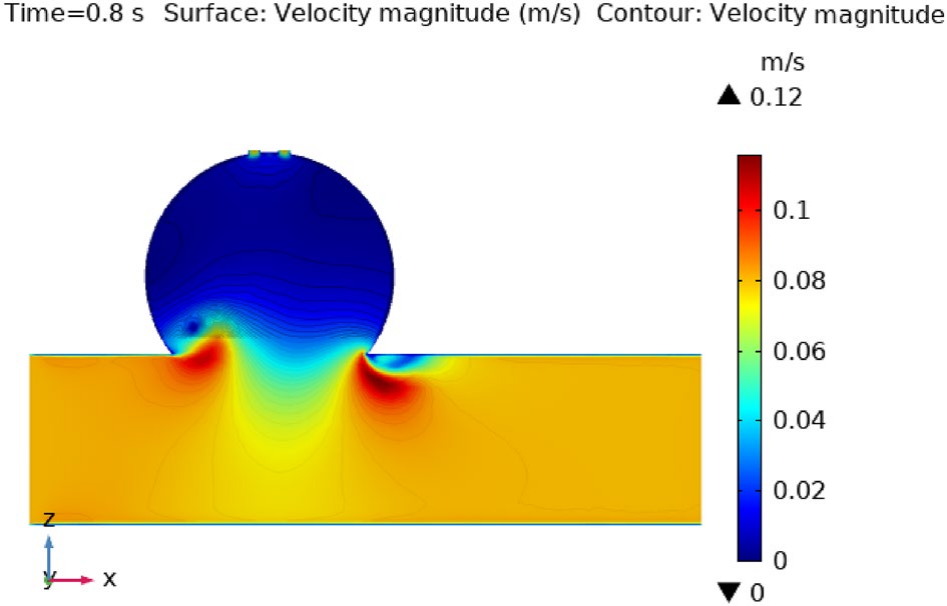
The velocity contour of the blood flow at time = 0.8 s.

**Figure 6:**
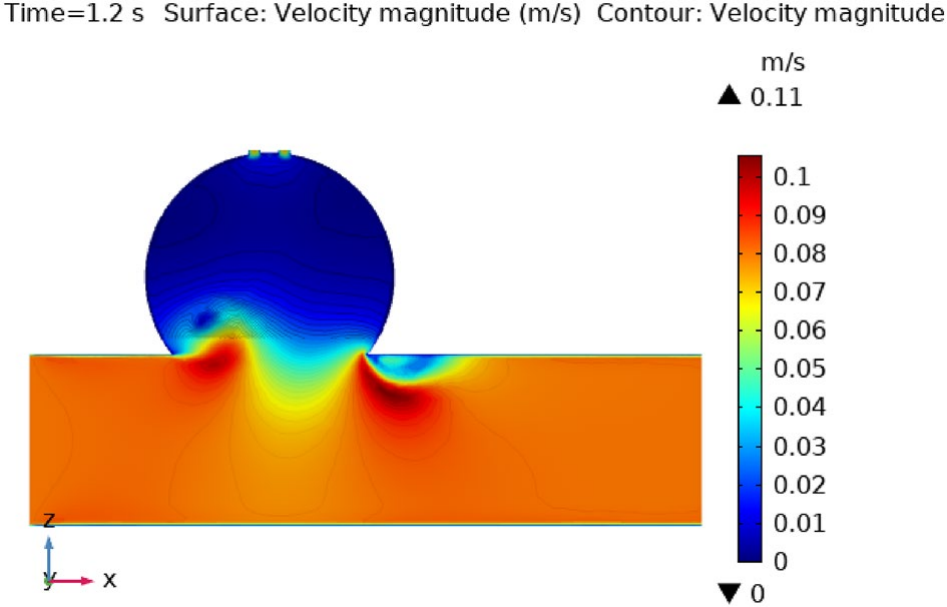
The velocity contour of the blood flow at time = 1.2 s.

**Figure 7:**
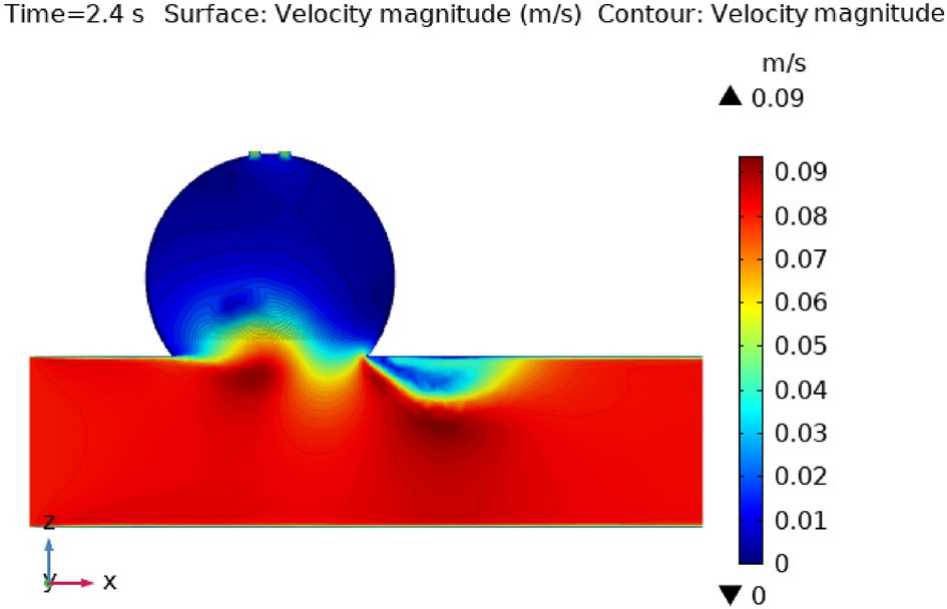
The velocity contour of the blood flow at time = 2.4 s.

Figures 3, 4, 5, 6 and 7 depicts the magnitude of the velocity contour profile implemented by hybrid nanoparticles in the $XZ$ cut plane at different time intervals of 0 s, 0.4 s, 0.8 s, 1.2 s, and 2.4 s. Figure 3 indicates that while the velocity contour is normal within the geometry, it is 2.02 ms^−1^ at the location where an aneurysm bursts and blood flowed from the dilatation. Figure 4, exhibits the blood’s velocity contour through the side wall ruptured dilatation at 0.4 s. At the aneurysm’s corner sites, where the side wall ruptured aneurysm broke and blood spilled from it, the blood flow’s highest velocity contour was recorded. The greatest velocity contour at this point is 0.13 ms^−1^ Nonetheless, at the border walls and within the broken aneurysm, the speed is at its nadir, with the minimum rate of speed being 0 ms^−1^. At moment 0.8 s, the speed abruptly decreases and comes to 0.12 ms^−1^ as represented in Fig. 5. The ruptured aneurysm’s interior and the artery’s outside margin have the lowest and highest velocities, respectively. Figure 6 exhibits that at time 1.2 s showing the same behavior as 0.8 s, but outing before and after an aneurysm velocity contour is higher than before. The greatest velocity magnitude is shown in Fig. 7 to be 0.09 ms^−1^ at t = 2.4 s illustrates a shift in maximum velocity contour during the whole length of the artery. The ruptured dilatation blood flow is at its lowest, while the fluid’s speed as it leaves the aneurysm and enters the burst zone is 0.03 ms^−1^. This result is consistent with the hypothesis that blood flows through the burst section increasing blood velocity and pressure on the artery walls. By incorporating these hybrid nanoparticles into the damaged artery, they create a scaffold that can reduce blood flow, enabling the artery to mend on its own. These Figs. 3, 4, 5, 6 and 7 show that velocity contour decreases in response to the amount of time elapsed, which is a result of the addition of silver and gold nanoparticles.

### Surface pressure contour profile

**Figure 8:**
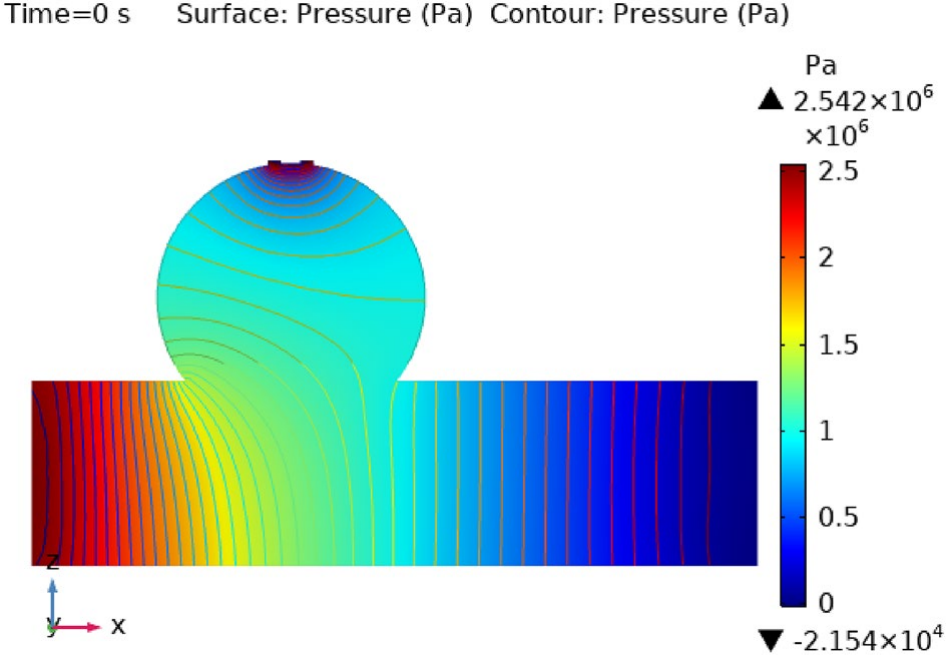
The pressure contour of the blood flow at time = 0 s.

**Figure 9:**
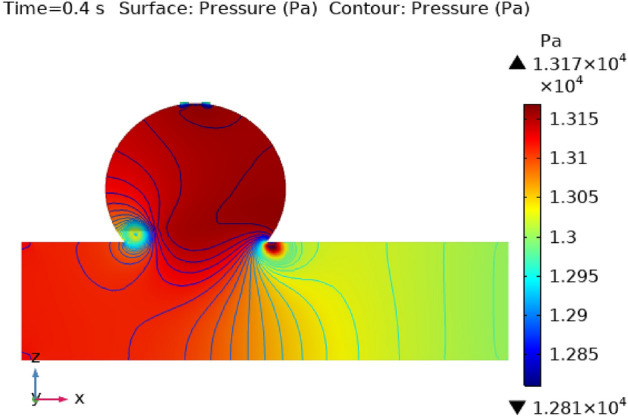
The pressure contour of the blood flow at time = 0.4 s.

**Figure 10:**
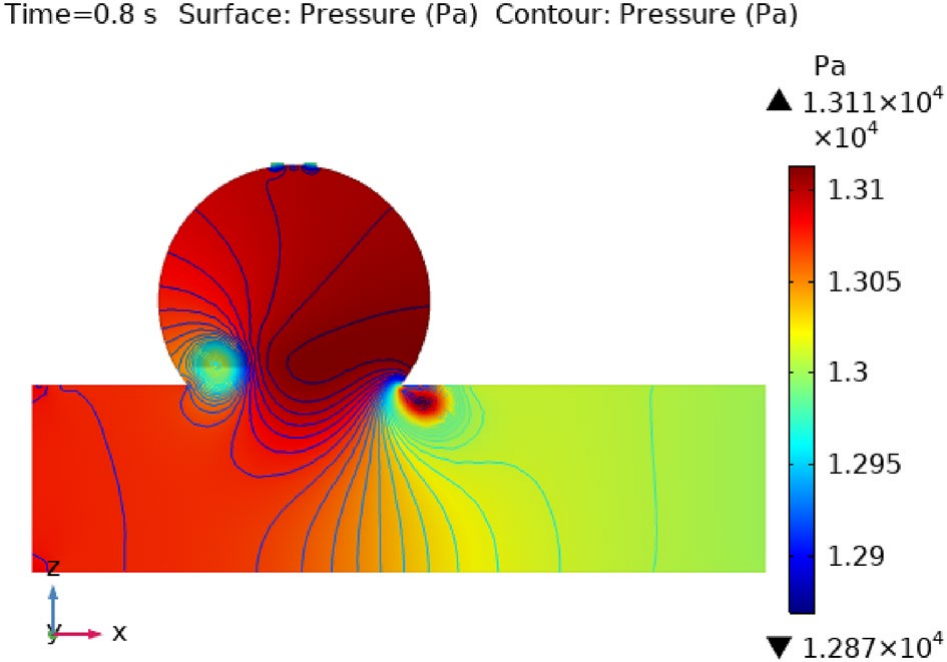
The pressure contour of the blood flow at time = 0.8 s.

**Figure 11:**
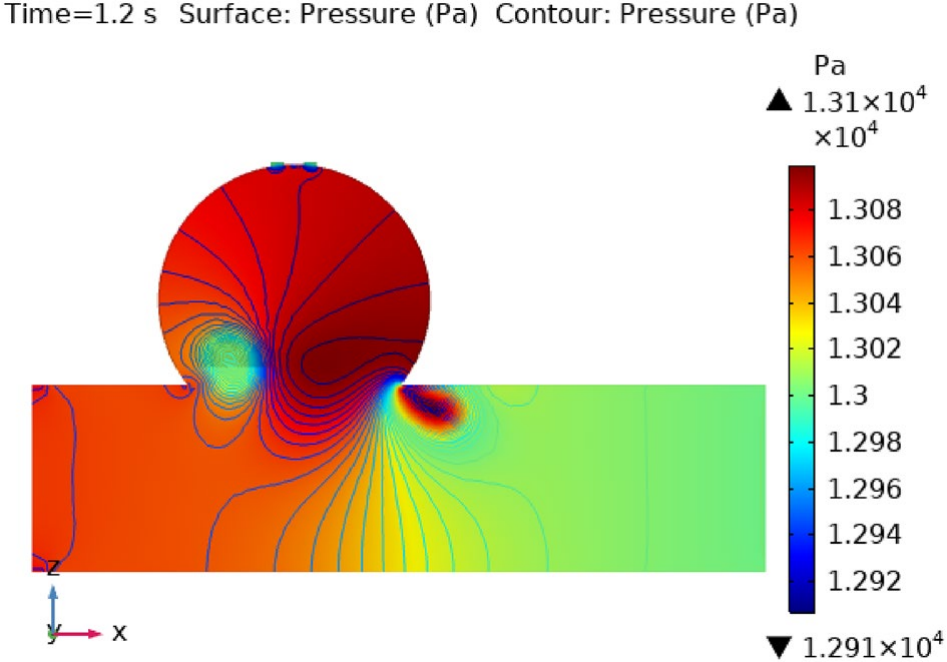
The pressure contour of the blood flow at time = 1.2 s.

**Figure 12:**
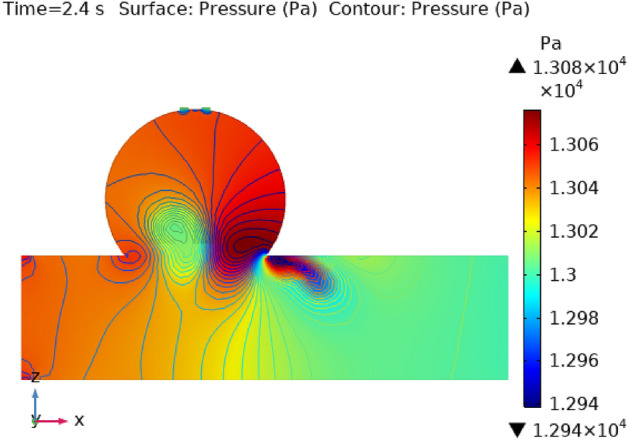
The pressure contour of the blood flow at time = 2.4 s.

Figures 8, 9, 10 and 11 use 3-D representations with durations of 0 s, 0.4 s, 0.8 s, 1.2 s, and 2.4 s to show the pressure contour intensity after injected nanoparticles. Figure 8 shows the pressure contour effort at a dilated arterial wall due to fluid activity for 0 s. The maximum contour pressure is 2.542 × 10^6^ pa at the inlet wall, and the minimum pressure is − 2.154 × 10^4^ pa at the outlet wall and the bursts area of dilatation. Figure 9 articulates the pressure contour distribution within the sidewall ruptured aneurysm at 0.4 s. In the aneurysm and the area where it burst, the pressure contour is now at its highest level of 13,170 Pa. The pressure contour at the point of the rupture of the artery is gradually abating as the blood is oozing out. The pressure contour is at its lowest point as the blood leaves the aneurysm, which is 1.281 × 10^4^ pa. The pressure contour inside the artery beyond the aneurysm ranges from 1.3 × 10^4^ to 1.305 × 10^4^ pa. The blood pressure distribution at time t = 0.8 s is shown in Fig. 10. The maximum and minimum pressure is 1.311 × 10^4^ pa and 1.287 × 10^4^ pa. The pressure contour variation at time t = 1.2 s is shown in Fig. 11. The lowest level of pressure contour along the artery is seen to occupy a bigger region than t = 0.8 s. The pressure contour difference is seen to peak at 1.31 × 10^4^ pa at the beginning of the creation of a shattered aneurysm and nadir at 1.2981 × 10^4^ pa when it overcomes all impediments at the aneurysm’s outflow. The peak and lowest pressures contour at time 2.4 s in Fig. 12, are 1.308 × 10^4^ pa and 1.294 × 10^4^ pa, respectively. The torn parts of the aneurysm trigger blood to seep; in this region and beyond the broken aneurysm all demonstrate the same pressure distribution of 1.301 × 10^4^ pa. Inside the broken aneurysm wall, limited space at the point where the aneurysm finishes a peak pressure is 1.308 × 10^4^ pa. Inside the broken aneurysm wall, a tiny area placed at the aneurysm’s endpoint has a high pressure contour of 1.308 × 10^4^ pa. These statistics imply that the addition of hybrid nanoparticles leads to a decline in pressure on the walls and a rise in blood circulation. The minimum and maximum values for all other points of time can be discerned from the legends and the pattern of pressure contour diagrams in all scenarios can also be seen. Each and every graph is symmetrical. Pressure contour readings vary according to position and according to time. All of these findings indicate that the inclusion of hybrid nanoparticles reduces pressure at the boundary and boosts blood flow.

### Temperature contour profile

**Figure 13:**
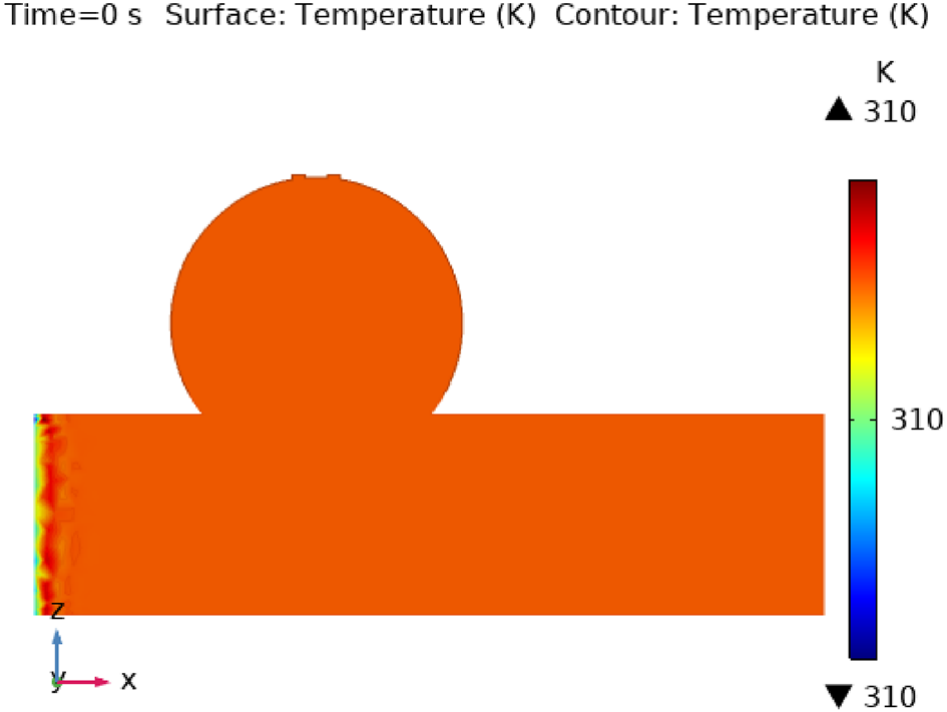
The temperature contour of the blood flow at time = 0 s.

**Figure 14:**
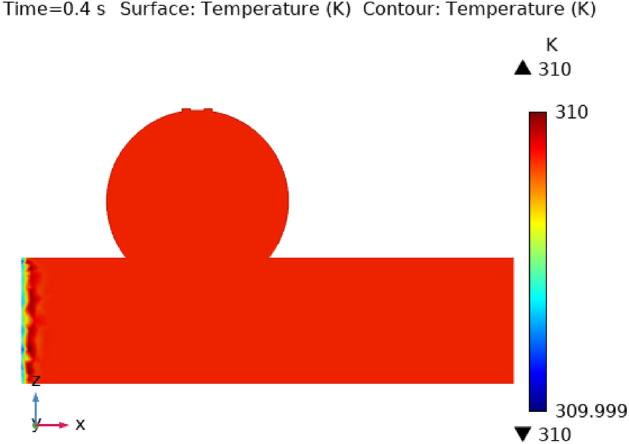
The temperature contour of the blood flow at time = 0.4 s.

**Figure 15:**
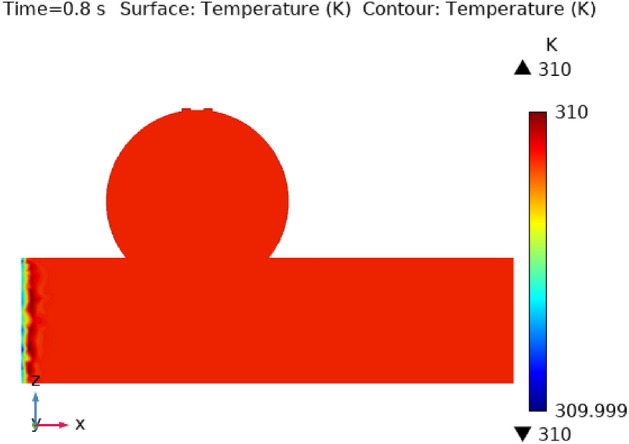
The temperature contour of the blood flow at time = 0.8 s.

**Figure 16:**
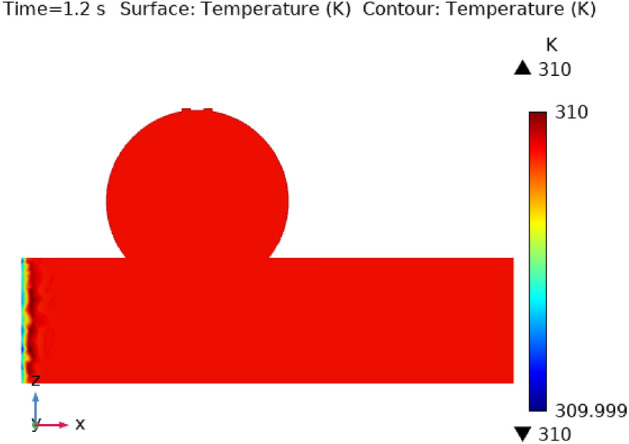
The temperature contour of the blood flow at time = 1.2 s.

**Figure 17:**
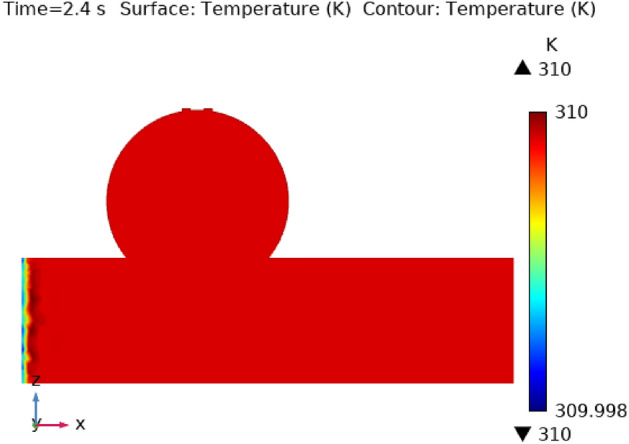
The temperature contour of the blood flow at time = 2.4 s.

Illustrations 13 through 17 use 3-D representations with durations of 0, 0.4, 1.2, 2.4, seconds to show the temperature intensity. Figure 13, shows that the temperature contour is constant in complete fig at times 0 s. Graph 14 shows that at t = 0.4 s, the temperature contour ranged from 309.99 to 310 K. While the rest of the artery has a constant temperature contour, the first portion of the artery has a varying temperature contour. Figures 14 and 15 shows that the temperature contour rises slowly in the little area at the beginning of the inlet. The maximum and minimum temperature contours are 310 K and 309.99 K, respectively, during a time of 0.8 s. Figures 16 and 17 illustrate the same temperature contour variation which is in Figs. 14 and 15. Temperatures contour in arteries have been stabilized using nanoparticles. They can absorb and expel heat energy, which aids in preserving a constant temperature contour. There is almost no variation in temperature contour over time.

## Illustration of line graphs

**Figure 18:**
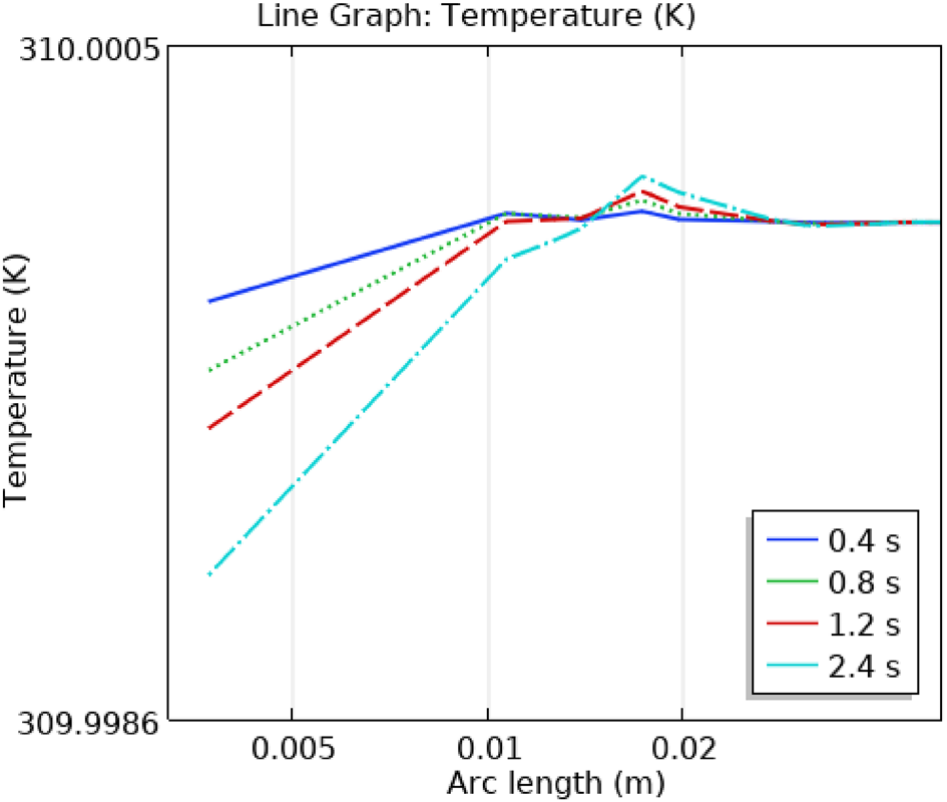
Line graph of blood flow temperature in artery.

Figure 18 shows the temperature profile in the artery as a function of time and location. The temperature starts low and gradually increases as the blood travels a certain distance. The graph shows that temperature changes over time as well. There is a spot where the temperature is the same all the time beyond a certain distance. After then, the temperature increases gradually before starting to gradually fall until it reaches a point where it is constant throughout time. The highest and lowest temperatures, despite the fact that they change throughout time, are 310.0005 k and 309.9986 k, respectively.

**Figure 19:**
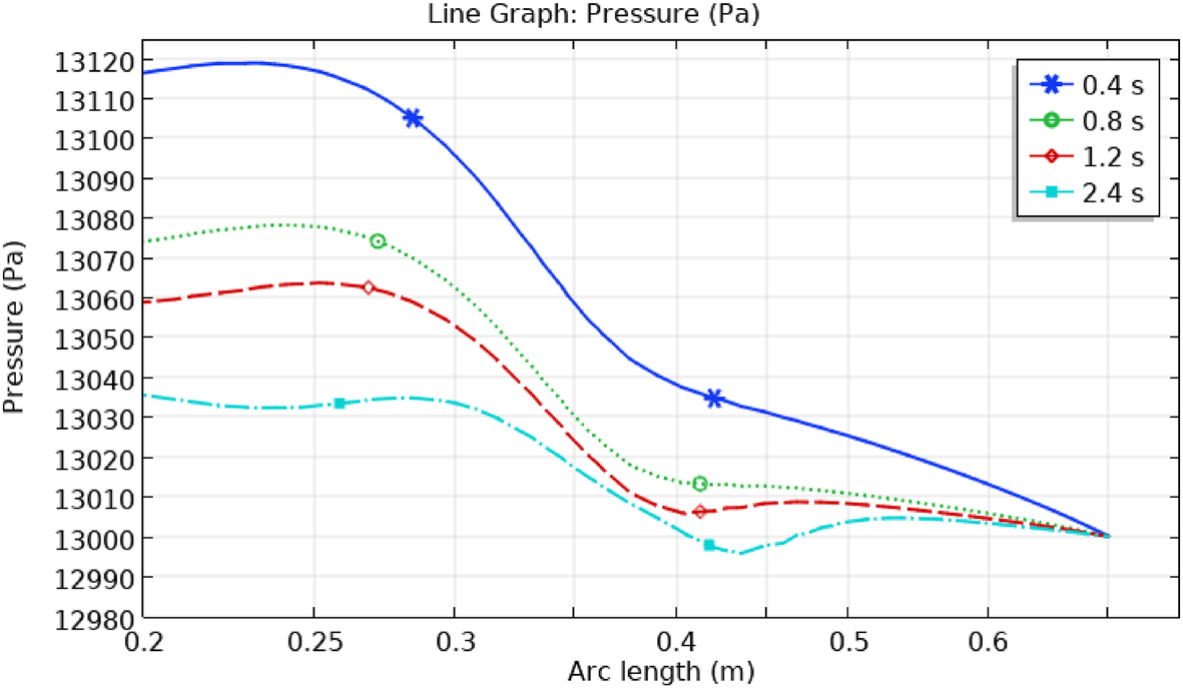
Line graph of blood pressure in artery.

Figure 19 shows the pressure gradient in the artery with the ruptured sidewall aneurysm. The first part of the artery has the highest pressure, which gradually decreases as it approaches the aneurysm. As the blood goes out, the pressure decreases. Time has an impact on the pressure as well, and the titles show how the pressure changes with time. As time goes on, the pressure decreases. The highest pressure is 13,120 pa, while the lowest is 12,990 pa.

**Figure 20:**
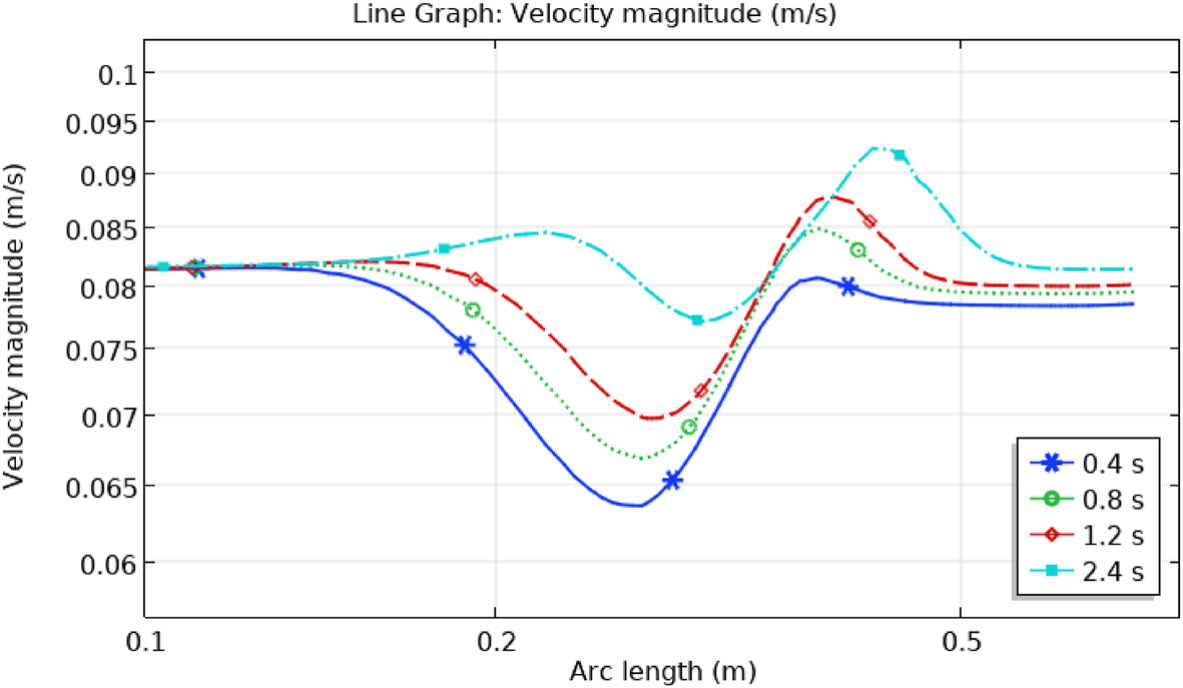
Line graph of blood flow velocity in artery.

The graph of velocity in the artery with the burst aneurysm is presented in Fig. 20 for the point and period. Initially, blood flows are usually in the artery due to the nonexistence of the burst sidewall aneurysm. As an aneurysm began at position x = 0.15 m, the highest velocity is 0.085 ms^−1^ for time 2.4 s and the lowest velocity is 0.065 ms^−1^for time 0.4 s. the overall maximum and minimum velocity is 0.094 ms^−1^ at 2.4 s and 0.065 ms^−1^ at 0.4 s.

## Conclusion

In order to examine the properties of blood flow instilling silver and gold hybrid nanoparticles in a side wall ruptured dilatation-affected artery, a mathematical and computational model is built in this work. The result is estimated numerically using the finite element technique (FEM), and blood possesses Newtonian characteristics. The main objective of this study is to summarize the CFD results for the velocity, temperature, and pressure over the damaged portion of the artery.

- Arterial diameter affects blood pressure, temperature, and flow rate.
- As time passed, the blood flow rate in this model reduced, although it was at its maximum during the initial moment when the aneurysm ruptured.
- The hybrid nanoparticles may absorb and reflect energy, which helps decrease pressure within the aneurysm and slow blood flow. Furthermore, the nanoparticles can operate as a scaffold to assist tissue regeneration after the rupture of a dilation artery and help the aneurysm to heal fast.
- To manage the aneurysm’s form and lower the chance of subsequent rupture and clotting, hybrid nanoparticles have also been applied.
- When an aneurysm ruptures, the pressure is at its highest and is concentrated inside the ruptured dilatation wall, while the pressure at the rupture site beyond the aneurysm is at its lowest. The afflicted artery’s pressure decreases over time using the hybrid nanoparticles helps to reduce the pressure at the artery wall.
- The early portion of the artery has a different temperature than the rest of the artery, which is constant. The increase in the volume percentage of hybrid nanoparticles lowers the temperature profile.
- Identification of potential risk factors, such as a narrow neck or an irregular shape, that may trigger rupture is made more accessible with the aid of 3D geometry of ruptured sidewall pathological aneurysms. By analyzing the 3D geometry of ruptured sidewall pathological dilatation, it is possible to uncover potential prognostic markers related to aneurysm rupture and make therapeutic decisions, such as when to undertake embolization or endovascular repair.

## Data Availability

All the data mentioned in this paper is included within the paper.
